# Disposition Kinetics of Levofloxacin in Sheep after Intravenous and Intramuscular Administration

**DOI:** 10.4061/2010/727231

**Published:** 2010-11-02

**Authors:** Ayman Goudah, Sherifa Hasabelnaby

**Affiliations:** ^1^Pharmacology Department, Faculty of Veterinary Medicine, Cairo University, Giza, P.O. Box 12211, Egypt; ^2^College of Pharmacy, Ohio State University, Columbus, OH 43210, USA

## Abstract

The present study was planned to investigate the disposition kinetics of levofloxacin in plasma of female native Barky breed sheep after single intravenous (IV) and intramuscular (IM) administration of 4 mg/kg body weight. The concentrations of levofloxacin in the plasma were measured using high-performance liquid chromatography (HPLC) with a UV detector on samples collected at 0, 0.08, 0.16, 0.33, 0.5, 1, 2, 4, 6, 8, 10, 12, 18, 24, 32, and 48 h after treatment. Following intravenous injection, the decline in plasma drug concentration was biexponential with half-lives of (*t*_1/2*α*_) 0.33 ± 0.12 h and (*t*_1/2*β*_) 3.29 ± 0.23 h for distribution and elimination phases, respectively. The volume of distribution at steady state *V*_(d(ss))_ was 0.86 ± 0.23 l/kg. After intramuscular administration of levofloxacin at the same dose, the peak plasma concentration (*C*_max_) was 3.1 ± 0.35 *μ*g/mL and was obtained at 1.64 ± 0.29 h (*T*_max_), the elimination half-life (*T*_1/2el_) was 3.58 ± 0.30 h, and AUC was 20.24 ± 1.31 *μ*g.h/mL. The systemic bioavailability was 91.35 ± 6.81 %. *In vitro* plasma protein binding was 23.74%. When approved therapy fails, levofloxacin may be used in some countries for therapy of food animals, however, that is not true in the US.

## 1. Introduction

Levofloxacin is a recently introduced third-generation fluoroquinolones with high activity against a wide spectrum of Gram-positive and Gram-negative bacteria [1]. In human clinical trials, levofloxacin has been found to be very effective in the treatment of infections of upper and lower respiratory tract, genitourinary system, and skin and soft tissue [2]. Compared to other fluoroquinolones, ofloxacin and ciprofloxacin, levofloxacin has more pronounced bactericidal activity against organisms such as *Pseudomonas* and *Enterobacteriaceae *[3]. The bactericidal effect of levofloxacin is achieved through reversible binding to DNA gyrase and subsequent inhibition of bacterial DNA replication and transcription [4–6]. Fluoroquinolones act by a concentration-dependent killing mechanism, whereby the optimal effect is attained by the administration of high doses over a short period of time [7]. This concentration-dependent killing profile is associated with a relatively prolonged postantibiotic effect [8]. For this class of antimicrobials, drug exposure, as measured by the area under the plasma concentration versus time curve (AUC), has been used to calculate surrogate efficacy indices, such as the AUC/MIC ratio, where MIC stands for the in vitro minimal inhibiting concentration of the tested bacteria [9–11]. Thus, variations of drug exposure can be associated with variations in the probability of a successful outcome with a specific dosage regime.

The drug undergoes a limited metabolism in rats and human [12] and is primarily excreted by kidney mainly as active drug. Inactive metabolites (N-oxide and desmethyl metabolites) represent <5% of the total dose [13], as other fluoroquinolones are metabolised in chickens as reported in [14–16], as chickens metabolized marbofloxacin to N-desmethyl-marbofloxacin.

The pharmacokinetics of levofloxacin has been investigated in a limited number of animal species including rats [17], rabbits [18], calves [19, 20], goats [21], cats [22], male camels [23], and stallions [24]. However, there is no available information on the kinetics of levofloxacin in the sheep. Therefore, the present study was undertaken to determine the disposition kinetics and bioavailability of levofloxacin in sheep following a single intravenous (IV) or intramuscular (IM) administration of 4 mg/kg bwt.

## 2. Material and Methods

### 2.1. Drugs and Chemicals

Levaquin (25 mg/mL of levofloxacin solution) was obtained from Janssen Pharmaceutica N V (Beerse, Belgium). Ciprofloxacin as internal standard was purchased from Sigma, Chemical Company (St. Louis, MO, USA). The solvents (Baker Inc., Phillipsburg, NJ, USA) used during the chromatographic analysis of the drug were HPLC grade.

### 2.2. Experimental Animals

The study was approved by the Animal Care and Use Committee at the Faculty of Veterinary Medicine, Cairo University. We used ten female native Barky breed 2-3 years old, 45–55 kg body weight. The animals were in optimal nutritional condition, fed on concentrated pellets, hay, and alfalfa, and had free access to water *ad libitum* daily. The health of all animals was monitored prior to and throughout the experimental period.

### 2.3. Drug Administration

The study was performed in two phases, following a crossover design (5 × 5). Animals were randomly assigned into two groups, with each group containing five animals. In phase one of the study, five animals were given a single intravenous injection into the left jugular vein at dose of 4 mg/kg bodyweight levofloxacin, and the other five were injected intramuscularly into the lower third region of the neck muscles with the drug at the same dose. Three ml venous whole blood samples were taken by jugular venepuncture into 10 mL heparinized Vacutainers (Becton Dickinson vacutainer Systems, Rutherford, NJ, USA). The sampling times were 0 (blank sample), 0.08, 0.16, 0.33, 0.5, 1, 2, 4, 6, 8, 10, 12, 18, 24, 32, and 48 h after treatment. All the blood samples were centrifuged at 3000 g for 15 min to separate the plasma. The plasma samples were frozen at −20°C until analysed. After a washout period of 2 weeks, the animals that had been injected intravenously with the drug were injected intramuscularly and vice versa. Pilot studies have shown that a 2-week period is enough to avoid carry over effect. Blood was collected and processed as above. The heparinized plasma samples were frozen at −20°C and analyzed by high-performance liquid chromatography (HPLC). All samples were analyzed within one week after each experimental phase.

### 2.4. Analytical Method

Plasma concentrations of levofloxacin were measured using a modified HPLC method [25]. Briefly, the HPLC system was performed on Shimadzu Liquid Chromatography System (Duisburg, Germany) equipped with an LC9A pump, an automatic sampler SIL6B, and a UV detector. Class LC 10 software version 1.6 (Shimadzu) was used for data analysis and processing. Levofloxacin and ciprofloxacin (as internal standard, 99.2 pure, 1 *μ*g 10/*μ*L methanol) were isolated from plasma. The plasma proteins were removed via methanol precipitation; 200 *μ*l plasma were mixed with 400 *μ*l methanol and vigorously shaken. The precipitated proteins were removed via centrifugation at 12000 × g for 5 min. Subsequently, 20 *μ*l of the supernatant were injected onto the column.

The HPLC separation was performed using a reversed-phase C_18_ column (Discovery, Supelco, 5 *μ*m, 4.6 mm × 150 mm) with an injection volume of 20 *μ*l. The mobile phase consisted of water : acetonitrile (80 : 20, v/v) with 0.3% of triethylamine and pH adjusted to 3.3 with phosphoric acid, using an isocratic form with a flow rate of 1.0  mL /min. The detector wavelength was set at 295 nm. The analytes were identified from the retention times of 97–99% pure reference standards. 

 The calibration curves of plasma were prepared with seven different concentrations between 0.01 and 10 *μ*g/*m* *L* using blank sheep plasma. A calibration curve was obtained by plotting the peak height ratio versus the nominal concentrations. The equation was calculated by the least-squares method using linear regression. The limit of quantification (LOQ) based on a signal-to-noise ratio >5 was 0.04 *μ*g/mL of levofloxacin in supplemented sheep plasma. Under our experimental conditions, the linearity of the method was from 0.01 to 10 *μ*g/mL of levofloxacin sheep plasma, and the value of correlation coefficients (*r*) was >0.99. The peak height ratios of an unknown specimen (peak height of levofloxacin/peak height of internal standard) were compared with that of the standard.

 The precision and accuracy of the method were evaluated by repetitive analysis of the plasma samples (*n* = 12) spiked with different known concentrations of levofloxacin. The percentage recoveries were determined by comparing the peak height of blank samples spiked with different amounts of drug and treated as any sample, with the peak height of the same standards prepared in phosphate buffer (*n* = 6). Intra-assay variations were determined by measuring six replicates (*n* = 6) of three standard samples used for calibration curves. The intra-assay variation coefficients were <4.3%. Interassay precisions were determined by assaying the three standard samples on three separate days. The Interassay variation coefficients were <4.6%. Recovery of levofloxacin from plasma was found to be 93%.

### 2.5. In Vitro Plasma Protein Binding

The extent of plasma protein binding was determined in vitro using ultrafiltration [26]; antimicrobial-free plasma from sheep fortified with known concentrations of levofloxacin (0.01, 0.16, 0.32, 1.25, 5, and 10 *μ*g/mL) was used. One ml of each sample was placed on a conditioned semipermeable membrane (Centriflow Cones CF-50, Amicon Corp., Lexington, MA, USA) resting on porous conical polyethylene support on the top of centrifuge tubes. The tubes were centrifuged at 1500 g for 45–60 min. Plasma samples and their corresponding ultrafiltrates were assayed by the same method (HPLC) as described above. The percentage of plasma protein binding was calculated according to the following equation:

(1) $$\begin{array}{cc} & \text{Protein}\;\text{binding}\;\text{\%}\\ & =\frac{\text{Total}\;\text{concentration}-\text{Ultrafiltrate}\;\text{concentration}}{\text{Total}\;\text{concentration}}\times 100. \end{array}$$

### 2.6. Pharmacokinetic Analysis

Pharmacokinetic analysis of plasma levofloxacin concentration versus time data was conducted by noncompartmental analysis using WinNonLin Professional version 4.1 software package (Pharsight Corporation, Mountain View, California). For the intravenous data, the appropriate pharmacokinetic model was determined by visual examination of individual concentration-time curves and by application of Akaike's Information Criterion (AIC) [27]. The plasma concentration-time relationship was best estimated as a two-compartment open model:

(2) $$C_{p}=Ae^{-\alpha t}+Be^{-\beta t},$$

where *C*_*p*_ is the concentration of drug in the plasma at time *t*, *A* and *B* are the zero-time drug intercepts of the distribution and elimination phase expressed as *μ*g/mL, *α* and *β* are the distribution and elimination rate constants expressed in units of reciprocal time (*h* ^−1^), and *e* is the natural logarithm base. The distribution and elimination half-lives (*t*_1/2*α*_ and *t*_1/2*β*_) were calculated according to standard equations [28], while the volume of distribution at steady state (*V*_(d(ss))_) and the mean residence time (MRT) were calculated according to the following equations (*V*_(d(ss))_ = Cl_*B*_ × MRT and MRT = AUMC/AUC), respectively. 

Following IM administration of levofloxacin, plasma concentrations data were analyzed by both compartmental and noncompartmental methods based on the statistical moment theory [28]. The terminal elimination half-life (*t*_1/2el_) and absorption half-life (*t*_1/2(*a*)_) were calculated as ln 2/*k*_el_ or ln 2/*k*_a_, respectively, where *k*_el_ and *k*_ab_ are the elimination and absorption rate constant, respectively. The areas under the concentration-time curves (AUC) were calculated by the trapezoidal rule and further extrapolated to infinity by dividing the last experimental plasma concentration by the terminal slope (*β*). The mean residence time (MRT) was calculated as AUMC/AUC, where AUMC is the area under the first moment curve, each individual curve of levofloxacin over time was analyzed to determine the peak concentration *C*_max_ (extrapolated from the curve), and the time to peak concentration *T*_max_ was read from the data. The systemic clearance was calculated as Cl = Dose/AUC. The absolute bioavailability (F%) was calculated as (AUC_IM_/AUC_IV_) × 100. In case of extravascular administration, the volume of distribution at steady state (*V*_(d(ss))_) and the systemic clearance (Cl_*B*_) were calculated according to the following equations (*V*_(d(ss))_ = Vd/F) and Cl_*B*_ = Cl_*B*_/F, respectively. 

 Pharmacodynamic efficacy of levofloxacin was determined by calculating the *C*_max_/MIC and AUC_24_/MIC ratios following IM administrations using the respective mean MIC value for susceptible *Klebsiella spp. *(0.06 *μ*g/mL), *Shigella* spp. (0.06 *μ*g/mL), *Salmonella* spp. (0.12 *μ*g/mL), *Proteus* spp. (0.06 *μ*g/mL), and *Acinetobacter* spp. (0.12 *μ*g/mL) according to Marshall and Jones [29]; these values were derived from those determined in the studies involving antibacterial activity of levofloxacin against strains isolated from human beings.

### 2.7. Statistical Analysis

The statistical analysis was performed using the SPSS 17.1 software package (SAS, Cary, NC, USA). Results are presented as arithmetic mean ± SD. The nonparametric Wilcoxon test was used to compare the parameters obtained after intravenous and intramuscular administration. Means were considered significantly different at *P* < .05.

## 3. Results

Clinical examination of all animals before and after each trial did not reveal any abnormalities. No adverse reactions were observed after the single-dose IV or IM administration of levofloxacin in the animals studied. Akaike's Information Criterion test indicated that a two-compartment model best represented the plasma concentration versus time data after IV administration of levofloxacin in sheep. 

**Figure 1:**
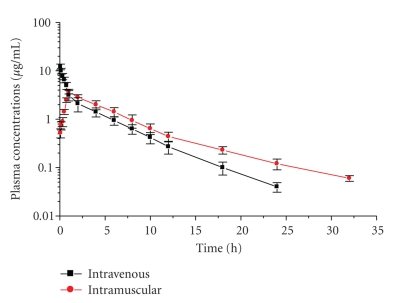
Mean ± SD plasma concentrations of levofloxacin in sheep after intravenous (■) and intramuscular (●) injection of 4 mg/kg b.wt. (*n* = 10).

 The mean plasma concentration-time profiles of levofloxacin following single IV and IM administrations of 4 mg/kg b.wt are presented graphically in Figure 1. Mean ± SD values of pharmacokinetic parameters estimated from the curve fitting are shown in Table 1. *In vitro* plasma protein binding of levofloxacin was 23.74%. Following intramuscular administrations of levofloxacin using MIC ≤ 0.12 *μ*g/mL, the *C*_max_/MIC_90_ ratio was 25.83-fold, and the AUC_0−24_/MIC_90_ ratio was 160.42 h.

## 4. Discussion

Plasma levofloxacin disposition curves after IV injection were best fit to an open bicompartmental model in all the animals, which is in accordance with the results reported for calves and lactating goats, respectively, [20, 21].

The *V*_(d(ss))_ is a clearance-independent volume of distribution that is used to calculate the drug amount in the body under equilibrium conditions [30]. Fluoroquinolones are lipid-soluble drugs that have a large volume of distribution [31]. The *V*_(d(ss))_ for levofloxacin was 0.86 l/kg in sheep indicating a relatively wide distribution after IV administration and it was slightly differing from that reported for levofloxacin in lactating goats 0.73 l/kg [21] and moxifloxacin in lactating goats (0.79 l/kg) [32]. The discrepancies between values calculated for pharmacokinetic parameters may be attributed to the animal species, the drug formulation employed, the age, size or sex of the animals, to differences in fatty tissue deposits between animal species or breeds, or even to interindividual variations and also due to the method of analysis of the drug [33].

The clearance of levofloxacin in sheep was 0.2 L/h.kg similar to those values reported in calves and lactating goats 0.19 and 0.18 L/h.kg [20, 21], respectively. 

The elimination half-life of levofloxacin following IV administration was 3.29 h. This value is close to that reported for levofloxacin in lactating goats 2.95 h [21] and longer than that reported in calves [20] 1.61 h, and shorter than that reported in rabbits 7.50 h [18].

Following intramuscular injection, the present data were best represented by a one-compartment model and the estimated *C*_max_ (3.10 *μ*g/mL) was similar to that data reported in calves and lactating goats (3.07 and 3.16 *μ*g/mL) [19, 21], respectively. 

The time to reach the maximum concentration of levofloxacin in sheep (*T*_max_ = 1.64 h) was longer than that recorded levofloxacin in calves (1.00 h) [19] and shorter than that reported in lactating goats 1.76 h [21]. The absorption process of levofloxacin was moderately fast as showed by the absorption rate constant (*k*_*a*_) 1.39 h^−1^, short absorption half-life (t_1/2(*a*)_) 0.51 h, and confirmed by the short *T*_max_ (1.64 h). The overall MRT was longer for IM administration compared with that for IV injection, with an estimated time of 5.33 h. This was expected as the MRT after IM injection depends on both the disposition and absorption rates. The MRT of levofloxacin was similar to that recorded for calves and lactating goats (5.57 and 5.24 h) [19, 21], respectively.

The elimination half-life value was 3.58 h after IM administration, similar to that recorded for calves and lactating goats (3.67 and 3.64 h) [19, 21], respectively.

The systemic bioavailability of levofloxacin in sheep after IM administration was 91.35%, and the absorption process was rapid with absorption half-life (*t*_1/2(*a*)_) 0.51 h. This value indicates the excellent absorption of the drug from that injection site. This value was similar to values reported for levofloxacin in lactating goats [21] and moxifloxacin in sheep [34] and that reported value was effectively higher than that reported for calves 56.6% [19]. 

Protein binding has long been considered one of the most important physicochemical characteristics of drugs, playing a potential role in distribution, excretion, and therapeutic effectiveness as a low protein binding generally enables a rapid and extensive distribution into the intracellular and extracellular space [35]. In this study, the *in vitro* plasma protein binding experiment showed that levofloxacin displayed a low level of binding to plasma proteins (approximately 23.74%) to sheep plasma. The results of *in vitro* protein binding may differ substantially depending on the methodology and experimental conditions [36]. The low protein binding of levofloxacin in sheep plasma proteins is in agreement with previously reported value of 22% in lactating goats [21], 24% in human [12] and 25% in rabbits [18]. Nevertheless, it was relatively lower to that reported (17%) in calves [19]. 

It has been established that for concentration-dependant fluoroquinolones, the AUC_0−24_/MIC_90_ ratio is the most important efficacy predictor, with the rate of clinical cure being greater than 80% when this ratio is higher than 100–125 [37]. A second predictor of efficacy for concentration dependent antibiotic is the ratio *C*_max_/MIC_90_, considering that values ≥10 would lead to better clinical results [38]. High *C*_max_/MIC_90_ ratios have been associated with a lower incidence resistance development [39]. It is suggested that the critical break points determining the efficacy of fluoroquinolones are *C*_max_/MIC_90_ ≥ 8–10, and AUC_0−24_/MIC_90_ ≥ 100 [38, 39]. The MIC of levofloxacin has not yet been determined for bacteria isolated from sheep. To cover most of the susceptible organisms, in this discussion, the MIC_90_ of 0.12 *μ*g/mL of levofloxacin has been taken into consideration [29]. Based on this data, a dosage of 4 mg/kg levofloxacin IM in sheep would result in a *C*_max_/MIC_90_ ratio of 25.83-fold, which exceeds the recommended ratio of 10. The second surrogate marker AUC_24_/MIC_90_ was 160.42 h. Based on the calculated *C*_max_/MIC_90_ and AUC_0−24_/MIC_90_, a dosage of 4 mg/kg b.wt. is recommended to treat infections caused by bacteria with MIC ≤ 0.12 *μ*g/mL.

It can be concluded that levofloxacin administered intravenously or intramuscularly in the applied dosing schedule is efficacious against bacteria with MIC ≤ 0.12 *μ*g/mL. Consequently, levofloxacin could be useful in the treatment of systemic infections in sheep after specific assessment of susceptible micro-organisms, also, when approved therapy fails, levofloxacin may be used in some countries for therapy of food animals, however, that is not true in the US. Only enrofloxacin is approved for beef cattle and cannot be used extralabel.

## Figures and Tables

**Table 1:** Mean ± *S* *D* plasma pharmacokinetic parameters of levofloxacin (*μ*g/mL) in sheep (*n* = 10) following IV and IM administration at a dose rate of 4 mg/kg b.w.

Parameters	Unit	IV	IM
*α*	h^−1^	2.19 ± 0.17	—
*k*_a_	h^−1^	—	1.39 ± 0.15
*t*_1/2*α*_	h	0.33 ± 0.12	—
*t*_1/2(*a*)_	h	—	0.51 ± 0.11
*β*	h^−1^	0.19 ± 0.09	—
*k*_el_	h^−1^	—	0.21 ± 0.04
*t*_1/2*β*_	h	3.29 ± 0.23	—
*t*_1/2el_	h	—	3.58 ± 0.30
*V*_(d(ss))_	l/kg	0.86 ± 0.23	1.02 ± 0.18
Cl_*B*_	L/h.kg	0.20 ± 0.05	0.19 ± 0.03
AUC	*μ*g.h/mL	21.61 ± 1.24	20.24 ± 1.31*
MRT	h	4.26 ± 0.94	5.33 ± 1.05*
*C*_max_	*μ*g/mL	12.17 ± 1.73	3.10 ± 0.35
*T*_max_	h	—	1.64 ± 0.29
*F*	%	—	91.35 ± 6.81

*β* (*k*_el_): elimination rate constant; *α* (*k*_*a*_): distribution (absorption) rate constant; *t*_1/2*α*_: distribution half-life; *t*_1/2(a)_: absorption half-life; *t*_1/2*β*_ (*t*_1/2el_): elimination half-life; *V*_(d(ss))_: volume of distribution; Cl_*B*_: total body clearance; AUC: area under the curve from zero to infinity by the trapezoidal integral; MRT: mean residence time; *C*_max_: maximum plasma concentration; *T*_max_: time to peak concentration; F(%): bioavailability; for IM, (*V*_(d(ss))_ = *V* *d*/*F*) and Cl_*B*_ = Cl_*B*_/*F*

*P < .05.
